# Robustness of CNN-augmented sequential models for Li-ion battery RUL prediction under data scarcity

**DOI:** 10.1371/journal.pone.0339528

**Published:** 2025-12-30

**Authors:** Jie Zhang

**Affiliations:** School of Business Administration, Capital University of Economics and Business, Beijing, China; The Hong Kong Polytechnic University, CHINA

## Abstract

Accurate Remaining Useful Life (RUL) prediction for Lithium-ion batteries is critical for system safety, yet its efficacy is frequently limited by data scarcity in industrial contexts. The robustness of hybrid architectures combining Convolutional Neural Networks (CNNs) with sequential models, a potential solution, has not been systematically evaluated. This study addresses this knowledge gap by first using a CNN to derive low-dimensional feature representations from full charge-discharge cycles. We then systematically assess the performance of five prominent sequential models (GRU, LSTM, Transformer, Neural ODE, and Transformer (Pre-LN)) on these features under progressively severe data scarcity (0%, 10%, 30%, and 50% cycle removal). Based on leave-one-out cross-validation on the NASA and CALCE datasets, the analysis demonstrates that the CNN-based feature extraction significantly enhances the robustness of all tested sequential models. Furthermore, recurrent networks such as GRU and LSTM, possessing strong sequential inductive biases, consistently outperform more complex architectures under data-constrained conditions. This research validates a robust predictive methodology and provides practical insights for developing reliable RUL predictors for industrial applications where data is sparse.

## Introduction

The high energy density, extended cycle life, and low self-discharge rate of Lithium-ion batteries (LIBs) have established them as the principal power source for a wide array of applications, from portable electronics to electric vehicles and grid-scale energy storage. Their deployment in safety-critical systems necessitates robust battery health management, wherein the accurate prediction of Remaining Useful Life (RUL) is paramount for ensuring operational safety, optimizing maintenance schedules, and determining second-life value. While data-driven deep learning methods have shown considerable promise in modeling complex degradation dynamics, their practical application is often impeded by data scarcity. Conventional models are typically developed assuming the availability of large, complete datasets. In industrial contexts, however, data is frequently sparse due to storage costs, intermittent sensor measurements, or the “cold-start” conditions of new equipment. Training deep neural networks under such constraints can lead to overfitting, which compromises generalization performance and model reliability. Consequently, a significant bottleneck remains in developing predictive models that maintain high accuracy under data-scarce conditions, hindering the translation of prognostic technologies from research to industrial practice. Some studies have begun to address this by developing data-driven methods based specifically on charging data from on-road vehicles [1].

The evolution of RUL prediction methodologies has seen a distinct shift from physics-based to data-driven paradigms. Early physics-based models, founded on equivalent circuits or electrochemical principles, offered high interpretability. Some approaches integrated physics-informed features to capture degradation across varied operational conditions, while others inferred degradation parameters linked to specific mechanisms, such as active material loss, to provide conservative RUL estimates [2–4]. With advancements in sensor and data storage technologies, data-driven methods became a primary research focus [5]. Initial techniques included two-stage prognostic methods employing health indicators and k-nearest neighbor classifiers, alongside ensemble algorithms that combined multiple models to improve predictive accuracy [6–8]. However, traditional statistical models have limitations in dealing with highly complex nonlinear processes such as battery degradation, so researchers have gradually turned to deep learning methods [9]. Furthermore, a significant body of research has focused on hybrid models to improve prognostic accuracy. These methods often integrate signal processing techniques, such as Variational Mode Decomposition (VMD), with deep learning models to separate global degradation trends from local fluctuations [10]. Other approaches combine probabilistic models, like Gaussian Process Regression (GPR) or Particle Filters (PF), to quantify uncertainty and fuse data from multiple sources [11,12]. Additionally, methods integrating optimization algorithms like Particle Swarm Optimization (PSO) with models such as Extreme Learning Machine (ELM) and Relevance Vector Machine (RVM) have been proposed to enhance feature selection and model parameter tuning [13]. The subsequent emergence of deep learning, particularly with architectures like Recurrent Neural Networks (RNNs) and their variants such as Long Short-Term Memory (LSTM) and Gated Recurrent Units (GRU), enabled effective modeling of long-term temporal dependencies inherent in battery aging [14,15]. To further refine performance, researchers have explored complex architectures incorporating self-attention mechanisms or hybrid models that fuse Temporal Convolutional Networks (TCNs) with GRUs to characterize both capacity regeneration and degradation trends [16].

To manage the high dimensionality of sensor data within each battery cycle, a two-stage architecture involving feature extraction followed by sequential modeling has become a dominant paradigm. One-dimensional Convolutional Neural Networks (1D-CNNs) are frequently employed as front-end feature extractors to automatically learn local, aging-related patterns from raw time-series data [17]. This module is typically combined with a back-end sequential model, with CNN-LSTM and CNN-GRU representing classic and effective pairings for capturing both intra-cycle local features and inter-cycle long-term dependencies [18–23]. Recent work has extended this paradigm by integrating signal processing techniques like CEEMDAN prior to a CNN-BiLSTM model or by developing CNN-Transformer architectures [24–27]. Further innovations in model architecture, such as lightweight deformable networks, have also been proposed to reduce computational cost for deployment on edge devices [28].

Despite these advances, the robustness of such models is severely challenged by data incompleteness. Methodologies for handling irregular time series may be categorized as either data-level imputation or model processing [29]. Imputation techniques, which range from simple interpolation to more complex methods, seek to fill missing values but risk introducing artifacts or bias [30]. In contrast, model approaches modify the architecture to intrinsically accommodate irregular data. A notable solution in this domain is the use of continuous-time models based on Neural Ordinary Differential Equations (Neural ODEs). These models, including the ODE-RNN, conceptualize the evolution of a system’s hidden state as a continuous dynamical process, enabling state estimation at any arbitrary time point and naturally handling asynchronous observations. This principle has been successfully applied to diverse fields such as human motion prediction and dynamic graph representation [31–35].

However, a critical gap persists in the validation of these advanced models under non-ideal data conditions. It is essential to distinguish between two forms of data incompleteness: observational irregularity, which involves missing data points within a cycle, and sample-level data scarcity, which refers to missing entire data cycles from a historical sequence. Although continuous-time models like Neural ODEs are designed to address the former, their efficacy in the latter scenario, a common industrial reality, has not been rigorously benchmarked against other sequential architectures [22]. Furthermore, the specific contribution of a CNN front-end to the overall robustness of a hybrid model under varying degrees of sample-level data availability has yet to be systematically quantified.

To address these unresolved questions, this study conducts a systematic and rigorous comparative analysis of the two-stage CNN-sequential hybrid framework. The objective is to establish fundamental design principles for developing RUL predictors resilient to the practical challenge of limited training data. Our contributions are as follows:

(1) We systematically validate the robustness conferred by the CNN front-end feature extractor, demonstrating its critical role in achieving high-accuracy RUL prediction with significantly reduced training samples.
(2) We quantitatively benchmark five representative sequential back-end models under multiple sample-level scarcity scenarios, revealing that recurrent networks with strong sequential inductive biases (GRU and LSTM) consistently outperform more complex architectures like the Transformer and Neural ODEs.
(3) We establish that aligning a model’s inductive bias with the physical nature of the data is more critical for robustness than architectural complexity alone. A direct comparison with a traditional physics-based feature engineering method (ICA) further confirms that the end-to-end data-driven paradigm offers superior resilience, thereby providing foundational principles for designing reliable prognostic systems in data-constrained environments.

The remainder of this paper is structured as follows. The Materials and methods section describes the methodology, including the datasets, data preprocessing pipeline, hybrid framework architecture, and the experimental design for simulating data scarcity. The Results section presents the comprehensive experimental results from quantitative and qualitative comparisons of the five sequential models. Subsequently, the Discussion section offers an in-depth analysis of the roles of inductive bias and feature extraction in model robustness, benchmarking the framework against a physics-based approach. Finally, the Conclusion section summarizes the key findings, contributions, limitations, and directions for future research.

## Materials and methods

### Problem definition

The fundamental prognostic task in this study is the prediction of Remaining Useful Life (RUL) for lithium-ion batteries from historical operational data. RUL is defined as the number of remaining charge-discharge cycles before a battery’s capacity degrades to a predefined End-of-Life (EOL) threshold, typically 80% of its nominal capacity. The prediction is based on a time-ordered sequence of multivariate observations captured during operation.

Formally, the complete operational history of a single battery is a sequence of feature matrices ($X=\left\{X_{1},X_{2},\ldots ,X_{N}\right\}$), where each $X_{c}\in {\mathbb{R}}^{d\times T}$ represents the data from the c−th cycle. Here, d is the number of sensor channels (e.g., voltage, current, temperature), and T is the number of time-steps within the cycle. At any given cycle c, the true RUL is $y_{c}=N_{EOL}-c$.

The primary challenge investigated is sample-level data scarcity, where entire cycles of data are missing from the historical record. Consequently, the model accesses a sparse, observed sequence $X_{obs}\subset X$. Our objective is to learn a robust mapping function, $f_{\theta }$, that takes a window of the most recent historical observations, however sparse, and predicts the current RUL. This can be expressed as:


$${\hat{y}}_{c}=f_{\theta }(X_{c-\omega +1},\ldots ,X_{c}),$$ (1)

where ω is the window size and ${\hat{y}}_{c}$ is the predicted RUL at cycle c. The function $f_{\theta }$, parameterized by our neural network model, is trained by minimizing the Mean Squared Error (MSE) loss function between the predicted and true RUL values across all available training instances:


$$L_{MSE}=\frac{\text{1}}{M}\sum_{i=1}^{M}{\left(y_{i}-{\hat{y}}_{i}\right)}^{2},$$ (2)

where M is the total number of training samples, $y_{i}$ is the true RUL for the i−th sample, and ${\hat{y}}_{i}$ is the model’s corresponding prediction.

### Datasets and preprocessing

#### Dataset description.

**Fig 1:**
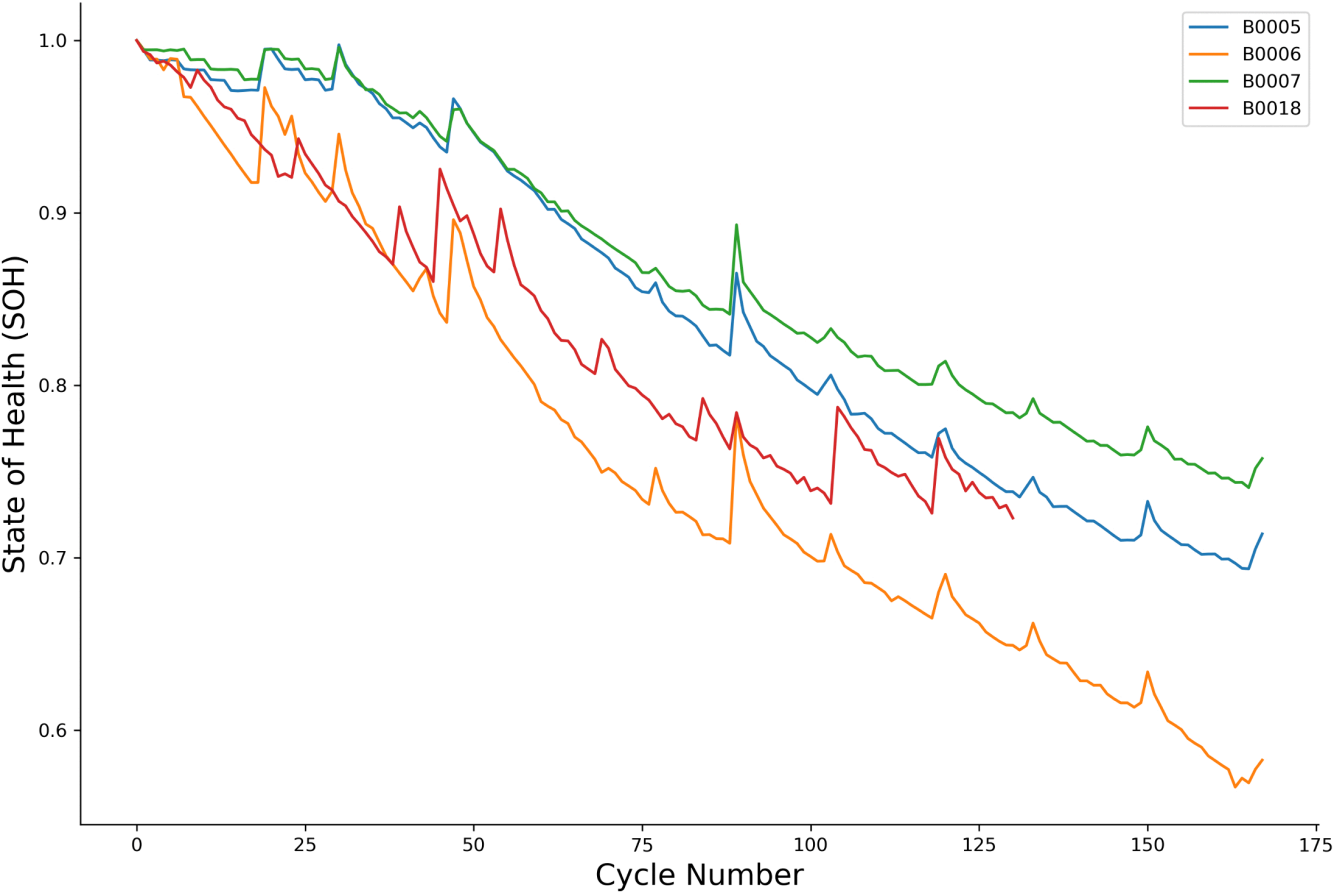
Capacity degradation curves for NASA batteries (B0005, B0006, B0007, B0018).

To ensure the generalizability of our findings, this study utilizes two widely recognized public benchmark datasets. The first is the NASA Prognostics Center of Excellence dataset, from which we selected four Lithium-ion batteries (B0005, B0006, B0007, and B0018). These batteries were subjected to a complete aging process under consistent laboratory conditions until their capacity degraded to the 80% EOL threshold. The raw data for each cycle includes time-series of voltage, current, and temperature, sampled at 1 Hz. The degradation patterns, characterized by non-linearity and periodic capacity recovery, are shown in Fig 1.

**Fig 2:**
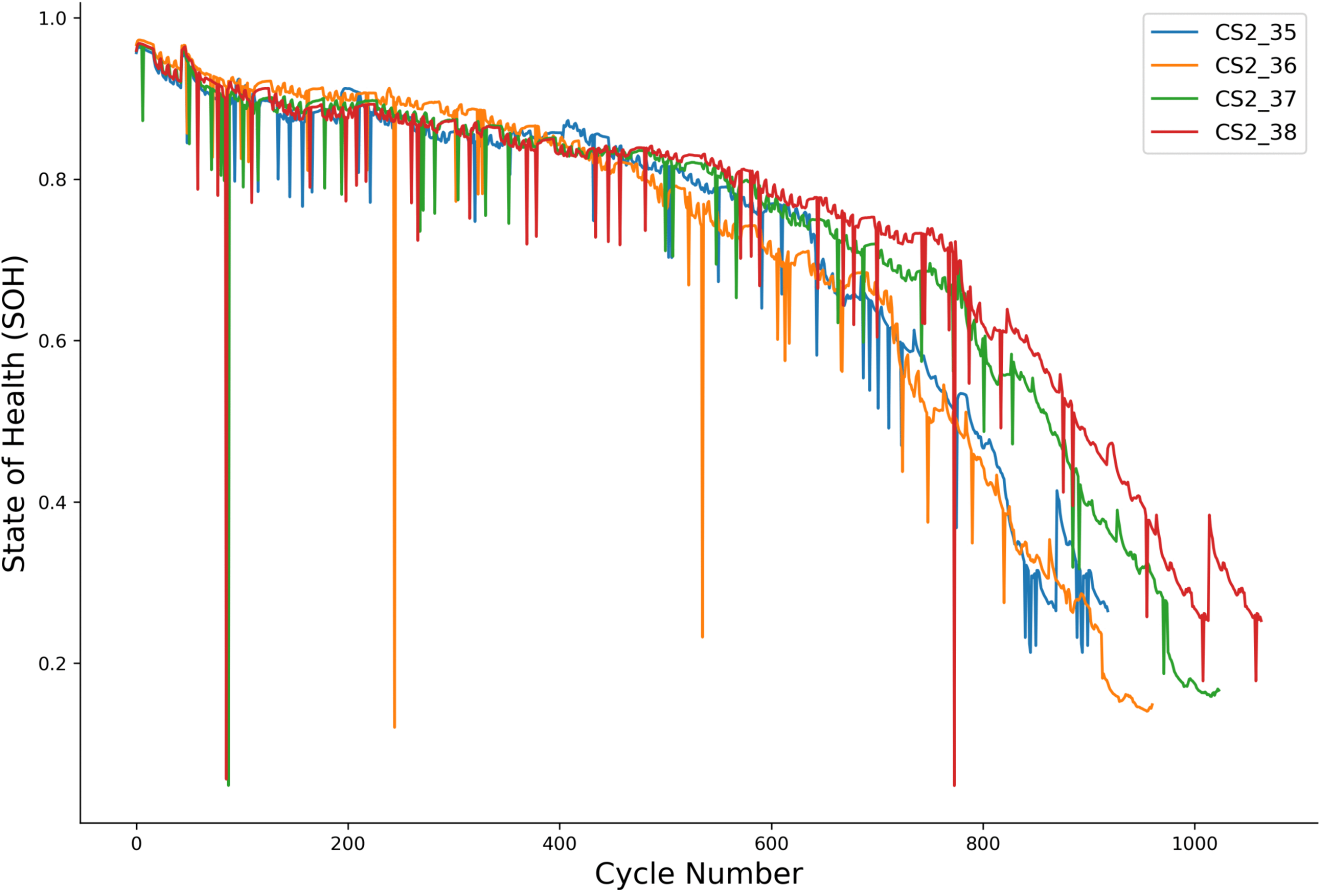
Capacity degradation curves for CALCE batteries (CS2_35, CS2_36, CS2_37, CS2_38).

The second dataset, from the Center for Advanced Life Cycle Engineering (CALCE) at the University of Maryland, includes four CS2 series batteries (CS2_35, CS2_36, CS2_37, and CS2_38). These batteries feature a different chemistry (Lithium Cobalt Oxide, LCO) and were subjected to distinct cycling protocols. A key distinction is the absence of temperature measurements; therefore, models were trained and evaluated on features derived exclusively from voltage and current data. The degradation patterns for these batteries, which exhibit a longer cycle life and more rapid capacity decay in later stages, are presented in Fig 2.

#### Data preprocessing pipeline.

A systematic preprocessing pipeline was implemented to convert the variable-length, raw time-series data from each charge cycle into uniform, fixed-size feature matrices. This process was applied consistently to all cycles and comprised three stages:

(1) Resampling to uniform length. To ensure a consistent input shape for the neural network, the time-series data for each charge cycle was resampled to a fixed length of 1000 data points using linear interpolation.
(2) Signal smoothing. A Savitzky-Golay filter (window size: 21, polynomial order: 2) was applied to the resampled voltage, current, and temperature sequences to mitigate high-frequency noise while preserving essential morphological features.
(3) Feature generation. To capture the dynamic characteristics of battery behavior, the first-order derivatives (rates of change) of the smoothed signals were computed and appended as additional feature channels.

For the NASA dataset, this pipeline resulted in a 6-channel feature matrix of shape (1000, 6) for each cycle. For the CALCE dataset, a 4-channel feature matrix of shape (1000, 4) was generated. This methodology ensures that each individual feature matrix is complete, allowing the study to focus specifically on the challenge of sample-level data scarcity.

### Experimental design

#### Overall research framework.

**Fig 3:**
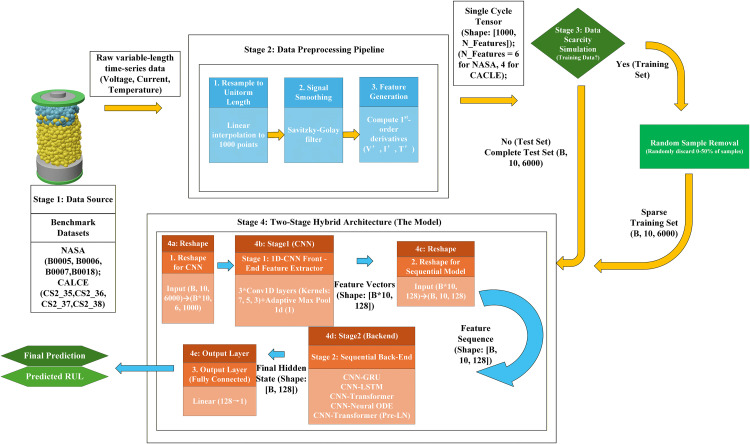
The overall research framework and data processing workflow.

The overall technical framework of this study, as illustrated in Fig 3, is a two-stage hybrid architecture designed to systematically evaluate the robustness of different sequential models under data scarcity. The framework consists of four main stages: (1) data sourcing, (2) a data preprocessing pipeline, (3) a data scarcity simulation applied only to the training set, and (4) the two-stage hybrid (CNN-Sequential) model used for prediction.

#### Simulation of data scarcity.

To systematically evaluate model robustness, two distinct scarcity scenarios were simulated:

(1) Sample-level scarcity. This scenario simulates the absence of complete historical records. For each cross-validation fold, a complete set of sliding window samples was generated from the training data1. This paper then simulated sample-level scarcity using a precise and reproducible protocol to ensure no bias was introduced. For a given scarcity rate (ρ), this paper performed a uniform random sampling without replacement on the indices of the training data instances. This was achieved by randomly shuffling the list of all training indices and selecting a subset corresponding to the desired removal percentage (e.g., 10%, 30%, or 50%). The samples corresponding to these selected indices were then discarded from the training set.

This specific protocol was chosen because it directly mimics two common industrial scenarios mentioned in our introduction: First, “intermittent sensor measurements”, where complete data records from certain cycles are sporadically lost. Second, the “cold-start” problem, where a new piece of equipment is deployed with only a sparse, non-continuous historical dataset. The test set for each fold remained complete and unchanged across all scarcity levels, enabling a rigorous evaluation of each model’s ability to generalize from sparse observations.

(2) Mixed scarcity. This supplementary experiment combined sample-level scarcity with observation-point irregularity. In addition to applying the same 0–50% sample removal rates to the training sets, a 30% observation-point irregularity was introduced to all data (both training and testing) by randomly setting 30% of the data points within each 1000-point cycle curve to zero. This protocol was designed to test the framework’s resilience to corrupted input signals.

#### Cross-validation scheme.

A Leave-One-Out Cross-Validation (LOOCV) scheme was adopted in all experiments and applied to both the NASA and CALCE datasets. For a given dataset, the LOOCV procedure involves distinct training and testing folds. In each fold, one battery was designated as the test set, while the remaining batteries were used for training. This process was repeated until every battery had served as the test set exactly once. To ensure the reproducibility of all experiments, including model initialization and data subsampling, a random seed of 42 was set.

### Model architectures

#### CNN front-end feature extractor.

A shared 1D-CNN was designed as the front-end to automatically extract health indicators from the high-dimensional time-series data of each cycle. The architecture processes an input tensor of shape (batch size, channels, 1000) and consists of three sequential Conv1d layers with 32, 64, and 128 output channels and kernel sizes of 7, 5, and 3, respectively.

This specific architecture was deliberately chosen to perform hierarchical multi-scale feature extraction, which is essential for capturing degradation-relevant patterns. In designing the hierarchical kernel, this paper adopts a pyramid structure where the kernel sizes decrease progressively (7→5→3). The initial, larger kernel (size 7) is designed to capture broad, low-frequency morphological shapes in the raw signal (e.g., the general slope of the voltage plateau). The subsequent, smaller kernels (sizes 5 and 3) then detect more intricate, high-frequency, and complex patterns from the features identified by the preceding layers.

Regarding the pooling strategy, the MaxPool1d layer (kernel size = 2) after the first two blocks has a dual function. They progressively downsample the sequence length (from 1000 points) and, crucially, provide local translational invariance. This invariance makes the features more robust to minor time-domain shifts in the signal, which are common in battery data. The final AdaptiveMaxPool1d (1) layer performs global max pooling. This is a critical step that forces the network to distill the single most salient activation from each of the 128 feature maps across the entire cycle’s temporal length.

The Tanh activation function is used. This entire process ensures the output is a fixed-length, 128-dimensional feature vector that represents a holistic signature of the cycle’s dynamics, rather than relying on a few isolated points. This vector serves as a single time-step input for the downstream sequential models.

#### Sequential modeling back-ends.

For each prediction, a window of 10 consecutive 128-dimensional feature vectors was fed into one of five sequential models. Key hyperparameters, including a hidden dimension of 128 and a learning rate of 0.0001, were kept consistent for a fair comparison. The supporting information (S2 File) provides the detailed mathematical formulas for these methods as well as a comprehensive list of all model hyperparameters and training configurations (S1 Table).

(1) CNN-GRU and CNN-LSTM. These models were implemented as representative recurrent neural networks (RNNs) with a strong sequential inductive bias. The architecture for both consists of a 2-layer GRU or LSTM network with a hidden dimension of 128 and a dropout rate of 0.1. The final RUL prediction is derived from the last hidden state via a fully connected layer.
(2) CNN-Transformer. A standard Transformer encoder architecture was implemented to evaluate the effectiveness of self-attention. Fixed sinusoidal positional encodings were added to the input sequence. The back-end consists of a 2-layer Transformer encoder with 4 attention heads and a hidden dimension of 128.
(3) CNN-Neural ODE. This model was implemented to handle irregular time series by conceptualizing hidden state evolution as a continuous-time process. An ODEFunc network parameterizes the derivative of the hidden state, which is integrated forward in time using the odeint function from the torchdiffeq library with the “dopri5” solver.
(4) CNN-Transformer (Pre-LN). A robust variant of the Transformer was included, which applies Layer Normalization before the multi-head attention and feed-forward sub-layers (Pre-LN) to improve training stability. Its structure mirrors the standard CNN-Transformer.

#### Evaluation metrics.

Model performance was evaluated using two standard regression metrics: Root Mean Square Error (RMSE) and Mean Absolute Error (MAE), calculated as:


$$RMSE=\sqrt{\frac{\text{1}}{M}\sum {\mathrm{\backslash nolimits}}_{i=1}^{M}{\left(y_{i}-{\hat{y}}_{i}\right)}^{2}},$$ (3)

$$MAE=\frac{\text{1}}{M}\sum {\mathrm{\backslash nolimits}}_{i=1}^{M}\left|y_{i}-{\hat{y}}_{i}\right|,$$ (4)

where M is the number of test samples, $y_{i}$ is the true RUL, and ${\hat{y}}_{i}$ is the model’s prediction. Additionally, to investigate the presence of systematic prediction bias, paired t-tests were conducted comparing the sequence of predicted RUL values against the true RUL values for each test battery. All modeling and analysis were performed using the PyTorch deep learning framework. All experiments were conducted on a workstation equipped with an NVIDIA RTX 4060 GPU and 32 GB of RAM. As indicated by our run logs, the average training time for a single cross-validation fold (100 epochs) on the CALCE dataset was approximately 5–6 minutes.

## Results

### Fragility of a direct sequential model

To motivate the adoption of a two-stage architecture, a preliminary experiment was conducted to assess the stability of applying a standard sequential model directly to high-dimensional features under data scarcity. A multi-layer LSTM network was configured to process the flattened, unprocessed 6000-dimension feature vector from each cycle. While the model could be trained with the complete dataset (0% sample removal), it exhibited severe numerical instability when sample-level scarcity was introduced. At a 10% sample removal rate, training runs frequently failed due to the generation of NaN (Not a Number) outputs. At higher scarcity rates (30% and 50%), successful completion of the training process was consistently unattainable. This outcome highlights the inherent difficulty that standard recurrent networks face when learning from high-dimensional, unprocessed sequences and provides a strong rationale for the proposed two-stage approach, which first extracts a robust, low-dimensional representation.

### Overall performance under sample-level scarcity

A comprehensive evaluation of the five hybrid architectures was conducted on the NASA and CALCE datasets under four levels of sample-level scarcity (0%, 10%, 30%, and 50% training sample removal). **Table 1** shows the aggregated performance metrics of all models.

**Table 1:** Average RMSE (MAE^*^) under different missing rates on NASA and CALCE datasets.

Model	Dataset	0%^a^	10% ^a^	30% ^a^	50% ^a^
CNN-GRU	NASA	0.046 (0.039)	0.044 (0.037)	0.039 (0.032)	0.044 (0.037)
	CALCE	0.039 (0.020)	0.042 (0.022)	0.042 (0.025)	0.042 (0.021)
CNN-LSTM	NASA	0.047 (0.040)	0.039 (0.033)	0.039 (0.032)	0.044 (0.038)
	CALCE	0.038 (0.018)	0.040 (0.020)	0.039 (0.020)	0.043 (0.025)
CNN-Transformer	NASA	0.065 (0.058)	0.057 (0.050)	0.053 (0.046)	0.061 (0.054)
	CALCE	0.042 (0.024)	0.050 (0.033)	0.050 (0.034)	0.046 (0.029)
CNN-Neural ODE	NASA	0.061 (0.053)	0.067 (0.057)	0.055 (0.045)	0.065 (0.056)
	CALCE	0.046 (0.023)	0.052 (0.034)	0.048 (0.025)	0.050 (0.027)
CNN-Transformer (Pre-LN)	NASA	0.054 (0.046)	0.060 (0.052)	0.067 (0.061)	0.055 (0.048)
	CALCE	0.040 (0.019)	0.042 (0.024)	0.041 (0.020)	0.043 (0.024)

*MAE values are listed in parentheses.

^a^The column headers 0%, 10%, 30%, and 50% represent the sample-level missing rate (ρ) applied to the training data.

As shown in **Table 1**, it reveals three primary findings. First, the CNN front-end conferred significant robustness against data scarcity to all downstream sequential models. Besides, the prediction error for all architectures remained highly stable across all scarcity levels on both datasets. For example, the RMSE for the CNN-LSTM model on the NASA dataset only ranged from 0.047 (0% removal) to 0.039 (30% removal) before increasing slightly to 0.044 (50% removal). This resilience was consistent on the CALCE dataset, demonstrating the framework’s generalizability. A secondary observation was that moderate scarcity appeared to have a regularizing effect; for instance, the CNN-GRU’s RMSE on the NASA dataset decreased from 0.046 to 0.039 when the sample removal rate increased from 0% to 30%.

Second, the recurrent networks with a strong sequential inductive bias (CNN-GRU and CNN-LSTM) outperformed other architectures. Under nearly all scarcity conditions across both datasets, these two models achieved lower RMSE and MAE values. Although the CNN-Transformer (Pre-LN) showed competitive performance on the CALCE dataset, the recurrent networks exhibited more stable performance compared to the standard Transformer and Neural ODE models. This indicates that the inherent sequential structure of RNNs is particularly suitable for characterizing the causal process of battery degradation.

Third, the two-stage framework demonstrated strong generalizability across the two distinct datasets. The better-performing models, CNN-GRU and CNN-LSTM, achieved a prediction accuracy on the CALCE dataset that was comparable to their performance on the NASA dataset. This suggests that the feature extraction and sequential modeling paradigm was not overfitted to a specific battery chemistry or operational protocol, which supports its robust application in diverse, real-world scenarios.

### Analysis of systematic prediction bias

To investigate whether the models exhibited a consistent directional error beyond the aggregate metrics, a statistical analysis for systematic prediction bias was performed. Paired t-tests were used to compare the sequence of predicted RUL values against the true RUL values for each test battery in the NASA dataset. And the results are detailed in **Table 2**.

**Table 2:** Paired T-test results for predicted and true RUL on the NASA dataset.

Model	Battery	0% ^a^	10% ^a^	30% ^a^	50% ^a^
CNN-GRU	B0005	15.472 (0.000^b^)	12.577 (0.000)	5.432 (0.000)	16.401 (0.000)
	B0006	−3.538 (0.001)	−3.856 (0.000)	−0.767 (0.444)	−1.103 (0.272)
	B0007	−4.676 (0.000)	13.567 (0.000)	1.395 (0.165)	−4.275 (0.000)
	B0018	−15.424 (0.000)	−14.464 (0.000)	−11.392 (0.000)	−14.340 (0.000)
CNN-LSTM	B0005	23.203 (0.000)	6.621 (0.000)	5.537 (0.000)	8.607 (0.000)
	B0006	−7.302 (0.000)	−13.361 (0.000)	−2.952 (0.004)	−4.650 (0.000)
	B0007	3.045 (0.003)	−0.787 (0.433)	5.910 (0.000)	−7.758 (0.000)
	B0018	−16.723 (0.000)	−12.100 (0.000)	−10.718 (0.000)	−15.731 (0.000)
CNN-Transformer	B0005	12.442 (0.000)	−3.516 (0.001)	8.922 (0.000)	13.916 (0.000)
	B0006	−17.414 (0.000)	−20.055 (0.000)	−20.252 (0.000)	−20.540 (0.000)
	B0007	−33.825 (0.000)	−16.501 (0.000)	−8.397 (0.000)	−11.294 (0.000)
	B0018	−21.273 (0.000)	−20.634 (0.000)	−14.529 (0.000)	−18.199 (0.000)
CNN-Neural ODE	B0005	6.775 (0.000)	5.073 (0.000)	7.715 (0.000)	20.412 (0.000)
	B0006	−21.969 (0.000)	−22.398 (0.000)	−10.987 (0.000)	−17.602 (0.000)
	B0007	11.276 (0.000)	13.506 (0.000)	5.590 (0.000)	3.587 (0.004)
	B0018	−16.826 (0.000)	−14.928 (0.000)	−11.199 (0.000)	−14.013 (0.000)
CNN-Transformer (Pre-LN)	B0005	14.704 (0.000)	29.739 (0.000)	26.806 (0.000)	21.544 (0.000)
	B0006	−12.812 (0.000)	−11.317 (0.000)	−24.160 (0.000)	−21.429 (0.000)
	B0007	−3.286 (0.001)	9.454 (0.000)	−5.407 (0.000)	0.075 (0.940)
	B0018	−18.341 (0.000)	−19.499 (0.000)	−22.316 (0.000)	−17.429 (0.000)

^a^The column headers 0%, 10%, 30%, and 50% represent the sample-level missing rate (ρ) applied to the training data.

^b^P values are in parentheses.

To assess whether the models exhibited a consistent directional error beyond the aggregate metrics, a statistical analysis for systematic prediction bias was performed. Paired t-tests were used to compare the sequence of predicted RUL values against the true RUL values for each test battery in the NASA dataset. The results, detailed in **Table 2**, show that the p-values were below the 0.05 significance level in the vast majority of test cases across all models and scarcity conditions. This provides statistical evidence that while the model predictions closely tracked the overall degradation trend, they contained a small but statistically significant systematic bias relative to the ground-truth curves. A closer examination of the t-statistics in **Table 2** reveals that the direction of this bias was often battery-specific. For instance, all models consistently produced positive t-statistics for battery B0005, indicating a systematic overestimation of its RUL across all scarcity levels. Conversely, the t-statistics for battery B0018 were uniformly negative, demonstrating a consistent underestimation of its RUL. For other batteries, such as B0007, the direction of the bias was less consistent and varied with the specific model and scarcity condition applied.

However, a few exceptions where no significant bias was detected were observed, primarily under conditions of moderate to high data scarcity. These included the CNN-GRU model’s predictions for battery B0006 at 30% (p=0.444) and 50% (p=0.272) scarcity, and the CNN-Transformer (Pre-LN) model’s prediction for battery B0007 at 50% scarcity (p=0.940). In these instances, the predicted RUL was not statistically distinguishable from the true values. This analysis confirms that while the models are highly accurate in terms of overall error, their predictions are not statistically unbiased. This finding underscores the critical distinction between model accuracy (low overall error) and model reliability (unbiased predictions), a particularly important consideration in safety-critical applications where even minor, systematic errors can have significant consequences.

### Visualization of prediction trajectories

**Fig 4:**
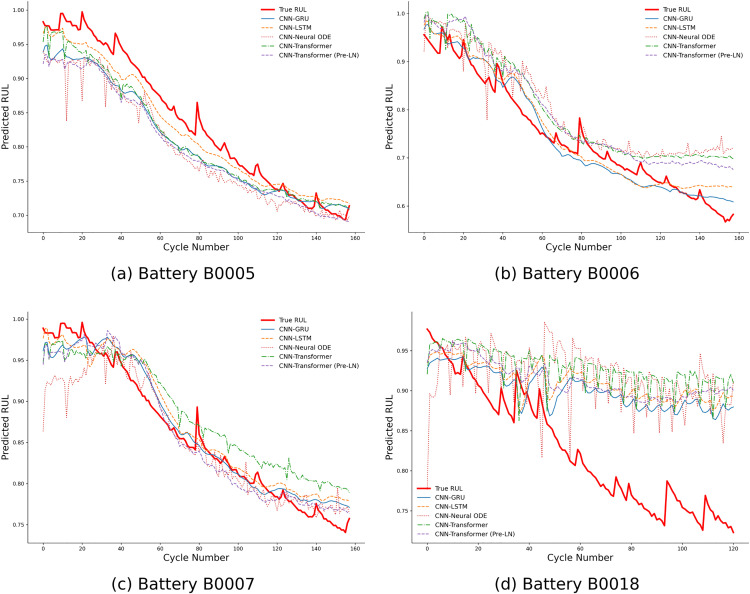
Comparison of RUL prediction curves for NASA batteries at 50% missing rate.

A qualitative assessment of the models’ dynamic prediction behavior was conducted by visualizing the predicted RUL trajectories against the true degradation curves. **Fig 4** presents this comparison for the most challenging condition, where models were trained with a 50% sample removal rate on the NASA dataset.

A consistent pattern emerges across all four test cases in **Fig 4**. The prediction trajectories of the CNN-GRU and CNN-LSTM models, which are based on recurrent architectures, closely and smoothly track the true RUL degradation curves. These models effectively capture the overall downward trend without exhibiting excessive local fluctuation, demonstrating high accuracy and stable prediction behavior even under severe data scarcity. In contrast, the other architectures exhibited more significant deviations from the ground truth. The CNN-Neural ODE model consistently produced predictions with pronounced high-frequency volatility across all test batteries, failing to generate a smooth degradation curve. Similarly, the attention-based models showed notable deviations. The standard CNN-Transformer and its Pre-LN variant, despite its architectural modification for improved stability, both exhibited significant prediction lag, particularly in the later stages of the batteries’ life. This lag is prominent for batteries B0005, B0006, and B0007 (**Fig 4 (a)**, **(b)**, and **(c)**), where their predictions are systematically higher than the true RUL. This performance difference is most starkly illustrated in the results for battery B0018, which features a more rapid degradation phase (**Fig 4 (d)**). In this critical scenario, both Transformer-based models were unable to capture the accelerated degradation trend, showing significant prediction lag and instability as their predicted RUL remained erroneously high while the battery was rapidly failing. In the same test case, the CNN-GRU and CNN-LSTM models continued to successfully track the actual RUL decline. This visual analysis provides strong qualitative support for the quantitative results, reinforcing the finding that the inherent sequential structure of recurrent networks provides a distinct advantage in prediction robustness, especially under challenging, data-scarce conditions.

### Performance under a mixed scarcity scenario

To provide a more comprehensive evaluation, a supplementary experiment was designed to test the models under a mixed scarcity scenario that combined both sample-level and observation-point data incompleteness. This protocol was intended to create a condition that aligns with the theoretical strengths of continuous-time models like the Neural ODE. The experiment introduced a 30% observation-point irregularity (by randomly setting 30% of data points to zero within each cycle curve) in addition to the 0–50% sample removal rates. The results of this experiment are presented in **Table 3**.

**Table 3:** RMSE comparison across original and mixed scarcity conditions (NASA dataset).

Model	0%	10%	30%	50%
CNN-GRU (Original ^a^)	0.046	0.044	0.039	0.044
CNN-GRU (Mixed scarcity experiment ^b^)	0.073	0.063	0.070	0.093
CNN-LSTM (Original)	0.047	0.039	0.039	0.044
CNN-LSTM (Mixed scarcity experiment)	0.071	0.083	0.084	0.083
CNN-Neural ODE (Original)	0.061	0.067	0.055	0.065
CNN-Neural ODE (Mixed scarcity experiment)	0.108	0.070	0.090	0.118

^a^Original refers to the baseline experiment simulating only sample-level scarcity, where 0–50% of entire training cycles were randomly removed.

^b^Mixed scarcity experiment refers to a scenario that combines the sample-level scarcity above with an additional 30% observation-point irregularity, created by setting 30% of data points within each cycle to zero for all data.

As seen in **Table 3**, the introduction of observation-point irregularity caused a significant increase in RMSE for all models compared to the original experiment, which confirmed that this type of data corruption poses a substantial challenge to the framework. For example, under the 50% sample removal condition, the RMSE for the CNN-GRU model increased from 0.044 in the original experiment to 0.093 in the mixed scarcity experiment. However, the experiment also yielded a key finding regarding the models’ relative resilience. Contrary to the theoretical expectation that the Neural ODE would be most robust in this scenario, its performance degraded more severely than that of the RNN-based models. At a 50% sample removal rate, the CNN-Neural ODE’s RMSE increased to 0.118, which was substantially higher than the 0.093 of CNN-GRU and 0.083 of CNN-LSTM under the same challenging conditions. This outcome does not refute the theoretical strengths of continuous-time models but instead exposes a critical architectural limitation of the proposed two-stage framework: the CNN front-end acts as a performance bottleneck. The standard 1D-CNN used in this study assumes a complete input sequence and its ability to extract meaningful patterns is compromised by corrupted inputs. Consequently, any downstream sequential model is fed a sequence of noisy feature vectors, preventing it from leveraging its core advantages.

### Comparison of data-driven and physics-based feature extraction

To evaluate the effectiveness of the proposed data-driven feature extractor, a direct comparison was made against a traditional physics-based feature engineering method using Incremental Capacity Analysis (ICA). Both feature sets were fed into the same GRU back-end model to ensure a fair comparison. The results, summarized in **Table 4**, demonstrate that the data-driven approach yielded superior performance across all conditions.

**Table 4:** Performance comparison of data-driven and physics-based feature extraction methods.

Feature Method	Sequential Model	0%^a^	50%^a^
Data-driven (CNN)	GRU	0.046^b^ (0.039^c^)	0.044 (0.037)
Physics-based (ICA)	GRU	0.070 (0.063)	0.066 (0.059)

^a^0%, 50% are missing rates. The column headers 0% and 50% represent the sample-level missing rate (ρ) applied to the training data.

^b^These values are RMSE.

^c^MAE values are listed in parentheses.

As shown in **Table 4**, with the complete training dataset (0% sample removal), the CNN-GRU model achieved an RMSE of 0.046 and an MAE of 0.039. In contrast, the model using ICA-based features recorded a significantly higher RMSE of 0.070 and an MAE of 0.063 under the same condition. This performance advantage was sustained under the high data scarcity scenario (50% sample removal), where the CNN-GRU model’s RMSE was 0.044 (MAE 0.037), compared to an RMSE of 0.066 (MAE 0.059) for the ICA-GRU model. These results indicate that the end-to-end, data-driven feature extraction framework is not only more accurate but also more robust than the traditional physics-based approach in data-constrained environments.

## Discussion

This study systematically evaluated a two-stage CNN-sequential framework for battery RUL prediction, with a specific focus on robustness under data scarcity. The primary findings were threefold. First, the CNN-based feature extraction conferred significant resilience against sample-level data scarcity to all sequential models tested. Second, recurrent architectures with a strong sequential inductive bias, namely GRU and LSTM, consistently outperformed more complex models like the Transformer and Neural ODE under these conditions. Third, this data-driven feature extraction paradigm demonstrated superior accuracy and robustness compared to a traditional physics-based feature engineering approach. The following sections will interpret these findings, discuss their implications, and contextualize them within the existing literature.

### The interplay of robustness and sequential inductive bias

A central finding of this research is that the architectural alignment between the model and the physical nature of battery degradation is critical for predictive robustness. The experimental results consistently showed that while the CNN front-end provided a universal baseline of robustness for all models, the structurally simpler GRU and LSTM back-ends outperformed the theoretically more powerful Transformer and Neural ODE models. This outcome arises from the interplay between the feature representation and the model’s sequential inductive bias. The front-end CNN module effectively transforms the high-dimensional raw data from each cycle into a low-dimensional, information-dense feature vector. This initial data compression creates a stable and robust representation of battery health, reducing the model’s sensitivity to the absence of subsequent training samples, as evidenced by the stable error metrics across increasing levels of data removal.

With this stable feature sequence as input, the inherent architecture of the back-end model becomes the primary determinant of performance. Recurrent neural networks like GRU and LSTM are explicitly designed to process information in an ordered manner. Their recursive mechanism, which updates a hidden state sequentially through time, naturally encodes the history of the degradation process. This structure is intrinsically aligned with the temporal and causal nature of battery aging, where the health state at one cycle is fundamentally dependent on the state of the previous cycle. In contrast, the Transformer’s self-attention mechanism processes all elements in a sequence in parallel, relying on external positional encodings to incorporate temporal information. While powerful for capturing complex, long-range dependencies, this capability appears less critical for the battery degradation process observed in this study, which is dominated by local, step-by-step transitions.

To empirically test the hypothesis that the Transformer’s high complexity, rather than its architectural bias, was the primary cause of this performance gap, we conducted a supplementary experiment. We evaluated a “Simple Transformer” (1 encoder layer, 2 attention heads, and a smaller 256-unit feed-forward network) against the original model (2 layers, 4 heads, 512-unit FFN) and the CNN-GRU. The results of this new comparison are presented in **Table 5**. First, the data shows that reducing complexity can mitigate overfitting, but its effect is not uniform. On the NASA dataset at 10% scarcity, the Simple Transformer (RMSE: 0.047) was a marked improvement over the Original (RMSE: 0.057). Second, on the CALCE dataset, the Simple Transformer (RMSE 0.044 at 30% scarcity) was consistently more robust than the Original (RMSE 0.050 at 30% scarcity). Third, despite these variations, the most critical and consistent finding is that the CNN-GRU model remained superior to both Transformer variants across all scarcity levels and on both datasets. Its performance was the most stable and accurate (NASA RMSE 0.039–0.046; CALCE RMSE 0.039–0.042). This new experiment confirms that while model complexity is a relevant factor, the inherent sequential inductive bias of the GRU architecture is the dominant contributor to its robust performance under data-scarce conditions.

**Table 5:** Performance comparison of CNN-Transformer-Simple and original models.

Missing Rate	Dataset	CNN-GRU	CNN-Transformer	CNN-Transformer-Simple
0% ^b^	NASA	0.046 ^a^ (0.039)	0.065 (0.058)	0.056 (0.049)
	CALCE	0.039 (0.020)	0.042 (0.024)	0.043 (0.026)
10%	NASA	0.044 (0.037)	0.057 (0.050)	0.047 (0.039)
	CALCE	0.042 (0.022)	0.050 (0.033)	0.046 (0.028)
30%	NASA	0.039 (0.032)	0.053 (0.046)	0.057 (0.049)
	CALCE	0.042 (0.025)	0.050 (0.034)	0.044 (0.024)
50%	NASA	0.044 (0.037)	0.061 (0.054)	0.061 (0.052)
	CALCE	0.042 (0.021)	0.046 (0.029)	0.045 (0.026)

^a^These values are average RMSE (MAE) under different missing rates on NASA and CALCE datasets.

^b^The column headers from 0% to 50% represent the sample-level missing rate (ρ) applied to the training data.s

Besides, it is important to clarify how models like the Transformer and Neural ODE handle the discrete nature of the cycle sequences. In our primary experimental setup (investigating sample-level scarcity), the input to the back-end model is a regularly-spaced sequence of 10 consecutive feature vectors. In this context, the Transformer and Neural ODE are not leveraging their specialized capabilities for temporal irregularity; they are functioning as general-purpose sequence-to-vector models, processing a discrete sequence of 10 steps. However, handling irregularity is highly relevant and was the precise motivation for our “Performance under a mixed scarcity scenario” experiment. In that supplementary experiment, we introduced observation-point irregularity by corrupting the input curves themselves (randomly setting 30% of data points to zero). The results of that experiment provided a critical insight: contrary to theoretical expectations, the CNN-Neural ODE’s performance degraded significantly. We concluded that this is due to the 1D-CNN front-end acting as a performance bottleneck. The standard CNN architecture assumes a complete, regular input curve and fails to extract meaningful features from corrupted, irregular data. Consequently, the downstream Neural ODE (or any other backend) receives a noisy, compromised feature sequence, preventing it from utilizing its architectural strengths. This remains a key limitation of the current two-stage framework, as noted in our conclusion.

However, this is not to dismiss the potential of the Transformer architecture. Its attention mechanism, which excels at identifying relationships across an entire sequence, may hold a distinct advantage when dealing with more complex and dynamic real-world data. Such data might contain abrupt capacity drops from specific events or require capturing ultra-long-range dependencies, such as linking an early-life operational anomaly to a much later, accelerated degradation phase. In these scenarios, the Transformer’s ability to look beyond immediate sequential steps could prove invaluable.

This suggests that for physical processes with strong temporal causality, a model’s architectural simplicity and alignment with the data’s nature are more important for achieving robust performance than its sheer complexity. It is important to note, however, that this conclusion is based on datasets collected under stable laboratory conditions; the performance hierarchy of these models might change under more dynamic, real-world operational scenarios.

### Comparison with SOTA methods

To benchmark the baseline performance of our framework, we compared our best-performing model (CNN-GRU) on the complete (0% missing) NASA dataset with several recent state-of-the-art (SOTA) methods. We acknowledge that direct comparisons are challenging, as studies may use different evaluation metrics (e.g., normalized RMSE vs. RMSE in percentage) or different prediction starting points. Our CNN-GRU model achieved an RMSE of 0.046 on the full NASA dataset. This performance is highly competitive. For example, Li et al. [10] proposed a complex hybrid approach (NGO-VMD-CNN-BILSTM) and reported an RMSE of 0.0076 on battery B0005 (starting at cycle 60). Wei et al. [36], using a VMD-GPR-GRU hybrid model, reported RMSEs as percentages, achieving 3.85% for a GRU model and 1.96% for their multi-scale model on battery B0005. Our baseline RMSE of 0.046 is well within the range of these advanced, optimized models.

Crucially, these SOTA studies primarily focus on optimizing prediction accuracy on complete datasets. They do not, however, systematically investigate the models’ robustness under the sample-level scarcity scenarios that are central to our work. While some recent studies have begun to address “limited data” or “missing data”, they often rely on interpolation rather than simulating the removal of entire historical samples. Therefore, our findings on the stability of CNN-augmented recurrent networks in data-scarce conditions provide a distinct contribution to the field.

### Comparison with physics-based feature engineering

The end-to-end, data-driven feature extraction employed in this study presents a clear alternative to traditional physics-based feature engineering methods, such as Incremental Capacity Analysis (ICA). While physics-based features offer high interpretability by linking metrics like ICA peak height to specific internal aging mechanisms of the battery, such as the Loss of Active Material (LAM) or Loss of Lithium Inventory (LLI), their effectiveness is often contingent on high-quality, complete data curves.

In contrast, the CNN front-end learns a holistic representation from the raw data. As suggested by our Grad-CAM analysis, rather than isolating a few predefined, interpretable points, the CNN builds a comprehensive degradation signature from the overall morphology of the entire charge cycle curve. The supporting information (S1 File and S1 Fig) presents the visualization results for the voltage and temperature curves of battery B0005 at early-life, mid-life, and late-life stages. This learned representation is inherently more resilient to noise and operational variations. Our direct comparative experiment supports this hypothesis. For the baseline, we extracted a 6-dimensional feature vector for each cycle from its ICA curve, comprising the voltage, height, and width of the two most prominent peaks. The CNN-GRU model achieved substantially lower prediction error than this ICA-GRU baseline, both with complete and sparse training data. Furthermore, the performance degradation of the CNN-GRU model was less pronounced as training samples were removed, indicating superior robustness. This result, as shown in Table 4, provides strong empirical evidence that for RUL prediction in data-constrained environments, the end-to-end feature learning paradigm offers a distinct advantage in both accuracy and robustness over traditional handcrafted feature engineering.

### The regularizing effect of sample-level scarcity

A counter-intuitive but consistent observation was that moderate levels of sample-level scarcity, specifically 10% and 30% sample removal, often resulted in improved generalization performance on the unseen test battery compared to training on the complete dataset. This phenomenon suggests that stochastic sample removal acts as an effective regularization mechanism.

Deep learning models are prone to overfitting when trained on small datasets, such as the ones used in this study. Training on the complete dataset allowed the models to memorize battery-specific artifacts, including stochastic capacity regeneration events, which are temporary capacity recoveries that do not reflect the underlying long-term degradation trend. Randomly removing a portion of the training samples likely pruned some of the most extreme instances of these non-monotonic dynamics. This process disrupts the model’s ability to memorize the training set, forcing it to learn a simpler and more generalizable representation of the fundamental degradation pattern. This finding highlights a critical principle in data-limited applications: preventing overfitting through implicit or explicit regularization is paramount for achieving robust generalization, and data quantity does not always equate to better model performance.

### Analysis and implications of systematic prediction bias

While the low overall error metrics (RMSE and MAE) confirm the models’ high accuracy, the paired t-test analysis revealed a statistically significant systematic bias between the predicted and true RUL values in most cases. This indicates that the prediction errors are not purely random and carry important practical implications, particularly for safety-critical systems.

This systematic deviation likely stems from two sources. First, the models may not fully capture all the complex, non-linear dynamics of battery aging, such as the aforementioned capacity regeneration phenomena, leading to localized periods of over- or underestimation. Second, the limited size and diversity of the training data may lead the model to learn degradation patterns that generalize well but are not perfectly calibrated to the unique trajectory of an unseen battery, resulting in a consistent offset.

The practical consequences of such bias are significant. A consistent overestimation of RUL could delay necessary maintenance and elevate the risk of unexpected failure, while a consistent underestimation could lead to the premature replacement of serviceable assets, incurring unnecessary costs. This distinction between model accuracy (low error) and model reliability (unbiased prediction) is crucial. Therefore, the industrial deployment of such a framework would necessitate a further calibration step to quantify this prediction bias and establish reliable confidence intervals for decision-making.

## Conclusion

This study addressed the critical challenge of data scarcity in predicting the Remaining Useful Life (RUL) for lithium-ion batteries by systematically evaluating a two-stage hybrid deep learning framework. The research was designed to establish robust design principles for developing prognostic models suitable for industrial contexts where historical data is often incomplete. The analysis has yielded several significant findings.

First, a 1D-CNN front-end proves to be a critical component for achieving robust RUL prediction under sample-level data scarcity. This module effectively reduces the complexity of the learning task by compressing high-dimensional intra-cycle data into information-dense feature vectors, thereby decreasing the data dependency of the downstream sequential models. Second, for degradation processes characterized by missing entire data cycles, aligning the model’s inductive bias with the physical nature of the process is paramount. Recurrent neural networks, specifically the GRU and LSTM architectures, consistently outperformed more complex models like the Transformer and Neural ODE. This result underscores the principle that for processes with strong temporal causality, a model’s structural alignment with the data’s underlying properties is essential for achieving superior performance and stability. Third, the framework’s robustness is specific to sample-level scarcity. The standard 1D-CNN architecture acts as a performance bottleneck when confronted with observation-point irregularity, which prevents downstream models from leveraging their intrinsic strengths.

From a practical perspective, these findings provide clear and actionable guidelines for engineering reliable RUL predictors for real-world applications where data collection may be intermittent. The validated two-stage framework offers a generalizable methodology that is not overfitted to a specific battery chemistry or operational protocol. Theoretically, this study contributes to the field by providing strong empirical evidence that a model’s inherent inductive bias is a more significant determinant of robustness than sheer architectural complexity, particularly in data-constrained scenarios.

Despite its contributions, this study has several limitations that should be acknowledged. The conclusions are drawn from benchmark datasets recorded under stable, laboratory-controlled operating conditions. This controlled setting was essential to rigorously isolate the impact of architectural inductive bias on robustness, excluding the interference of external noise. Specifically, the NASA and CALCE datasets involve single-cell tests under consistent, full charge-discharge cycling protocols and stable temperatures. Real-world applications, particularly in electric vehicle (EV) battery modules, introduce far greater complexity. These complexities include:

First, dynamic operational stress. EV operation is characterized by partial and irregular charge-discharge cycles, highly variable current rates, and fluctuating environmental temperatures, which are not present in these datasets. Second, cell-to-module dynamics. These findings are based on single cells. An EV battery module consists of numerous cells connected in series and parallel. The module’s overall degradation and RUL are governed not only by individual cell aging but also by cell-to-cell variations and imbalances, which introduce failure modes not captured by single-cell data.

Therefore, while this study establishes the robustness of RNNs under sample-level scarcity in a controlled setting, the direct universality of these findings (or the ranking of model performance) is not guaranteed for complex, multi-cell systems under dynamic operational stress. Besides, and most critically, the proposed framework relies on a preprocessing pipeline that assumes the availability of complete, consistent charge-discharge cycles. Our 1D-CNN front-end requires each cycle to be resampled to a fixed-length (1000-point) vector. This assumption does not hold in real-world electric vehicle (EV) applications, which are dominated by partial charging protocols (e.g., charging from 30% to 70%) and highly variable (non-constant) discharge currents. Our current method cannot process this type of fragmented and variable-length data. Therefore, the direct application of this specific framework to on-road EV data requires further adaptation.

Future research should be directed toward overcoming these limitations. First, a crucial next step is to validate the proposed framework on datasets that include dynamic operational loads and fluctuating environmental conditions to ensure real-world applicability. Second, to address the bottleneck identified by our mixed-scarcity experiment, future work should focus on designing novel front-end architectures that can natively handle observation-point irregularity, specifically Transformers applied directly to raw variable-length data or graph-based extractors. Finally, as the current framework provides deterministic point predictions, extending it to provide reliable uncertainty quantification (e.g., through Bayesian neural networks or deep ensembles) is a critical direction for enhancing its utility in safety-critical decision-making.

## Supporting information

S1 Fig This is the Fig A1. Cross-cycle Grad-CAM saliency map visualization (B0005).
(TIF)

S1 Table This is the Appendix C: Hyperparameter configuration for all models. (DOCX)

S1 File This is the Appendix A: Saliency map visualization of the CNN feature extractor. (DOCX)

S2 File This is the Appendix B: Mathematical formulations of model architectures. (DOCX)
