# Supplementary material for: Robustness of CNN-augmented sequential models for Li-ion battery RUL prediction under data scarcity
Source: PLoS One. 2025 Dec 30;20(12):e0339528. doi: 10.1371/journal.pone.0339528 (PMC12752999; doi:10.1371/journal.pone.0339528)
Supplement: S1 Table — (DOCX) [file pone.0339528.s002.docx]

# **Appendix C:** **Hyperparameter configuration for all models**

**Table C 1** is the hyperparameter configuration for all models.

**Table C 1. Hyperparameter configuration for all models.**

| Parameter Category | Hyperparameter | Value |
| --- | --- | --- |
| Data Preprocessing | Resample Points | 1000 |
|  | Window Size (Sequence Length) | 10 |
|  | N_Features (NASA) | 6 |
|  | N_Features (CALCE) | 4 |
| Training (Shared) | Epochs | 100 |
|  | Batch Size | 32 |
|  | Learning Rate | 0.0001 |
|  | Optimizer | Adam |
|  | Loss Function | Mean Squared Error (MSE) |
|  | Random Seed | 42 |
| CNN Front-End | Layer 1 | Conv1d (in=N_Features, out=32, kernel=7, padding=3), Tanh, MaxPool1d(kernel=2) |
|  | Layer 2 | Conv1d (in=32, out=64, kernel=5, padding=2), Tanh, MaxPool1d(kernel=2) |
|  | Layer 3 | Conv1d (in=64, out=128, kernel=3, padding=1), Tanh |
|  | Pooling | AdaptiveMaxPool1d (1) |
|  | Output Dimension | 128 (CNN_OUT_DIM) |
| Sequential Back-End | Hidden Dimension (Shared) | 128 |
|  | Dropout Rate (Shared) | 0.1 |
|  | CNN-GRU / CNN-LSTM | N_Layers = 2 |
|  | CNN-Transformer (Original) | N_Layers = 2, N_Heads = 4 |
|  | CNN-Transformer (Simple) | N_Layers = 1, N_Heads = 2 |
|  | CNN-Transformer (Pre-LN) | N_Layers = 2, N_Heads = 4 |
|  | CNN-Neural ODE | Solver = 'dopri5' |
