# Supplementary material for: Robustness of CNN-augmented sequential models for Li-ion battery RUL prediction under data scarcity
Source: PLoS One. 2025 Dec 30;20(12):e0339528. doi: 10.1371/journal.pone.0339528 (PMC12752999; doi:10.1371/journal.pone.0339528)
Supplement: S1 File — (DOCX) [file pone.0339528.s003.docx]

# Appendix A: Saliency map visualization of the CNN feature extractor

To investigate which features the CNN front-end model focused on, Grad-CAM was employed to generate saliency maps for the input signals. **Fig A 1** presents the visualization results for the voltage and temperature curves of battery B0005 at early-life, mid-life, and late-life stages. The analysis revealed that the model learned a holistic representation from the overall morphology of the input curves rather than focusing on specific, isolated points.


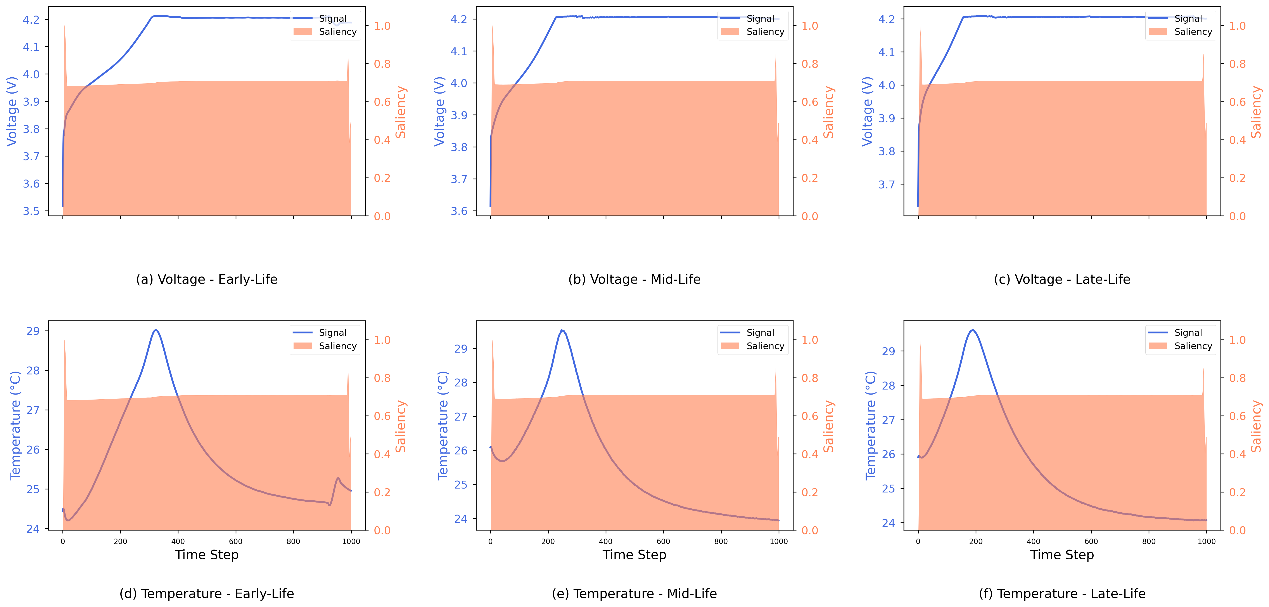


**Fig A 1. Cross-cycle Grad-CAM saliency map visualization (B0005). Contains detailed model implementations, hyperparameter configurations, feature descriptions, and supplementary figures including Grad-CAM visualizations.**

As shown in **Fig A 1**, the saliency attributed to the voltage signal was consistently high and uniformly distributed across the entire charging phase. A similar pattern was observed for the temperature signal (**Fig A 1** (d)-(f)), where the model assigned high importance to the complete thermal behavior curve. It is noteworthy that this pattern remained stable from the early-life to the late-life stages of the battery. This consistency indicates that the feature extractor adopted a robust strategy for identifying degradation-related patterns throughout the battery’s lifecycle. These findings suggest that the data-driven feature extractor builds a comprehensive degradation signature from the entire cycle, which supports the method’s overall robustness.
