# Supplementary material for: Robustness of CNN-augmented sequential models for Li-ion battery RUL prediction under data scarcity
Source: PLoS One. 2025 Dec 30;20(12):e0339528. doi: 10.1371/journal.pone.0339528 (PMC12752999; doi:10.1371/journal.pone.0339528)
Supplement: S2 File — (DOCX) [file pone.0339528.s004.docx]

# **Appendix B: Mathematical formulations of model architectures**

This appendix provides the detailed mathematical formulations for the key neural network architectures evaluated in this study.

## **Convolutional Neural Network (CNN)**

A 1D-CNN is used for feature extraction. Its primary operations are convolution and pooling.

(1) Convolutional Layer. This layer applies a set of learnable filters (kernels) across an input sequence to detect local patterns. The operation to produce the$j-th$ feature map$y_{j}^{l}$ at layer$l$ from the input $x_{i}^{l-1}$ is defined as:

$y_{j}^{l}=\sigma(b_{j}^{l}+\sum_{i} k_{ij}^{l}*x_{i}^{l-1})$,

where denotes the convolution operation,$k_{ij}^{l}$ and $b_{j}^{l}$ are the weight matrix and bias for the$j-th$ kernel, and $\sigma$ is a non-linear activation function.

(2) Pooling Layer. This layer downsamples the feature maps to reduce dimensionality and provide a degree of translational invariance. Max Pooling selects the maximum value within a sliding window $W_{n}$:

$p_{j}(n)=\max_{m\in W_{n}} y_{j}(m)$,

where $p_{j}(n)$ is the output of the pooling operation for the window $W_{n}$.

## **Long Short-Term Memory (LSTM)**

LSTM is an RNN variant designed to capture long-term dependencies via a gating mechanism that controls a memory cell. For a given time step $t$, with input $x_{t}$ and previous hidden state $h_{t-1}$, the updates are as follows:

(1) Forget Gate ($f_{t}$). Determines which information to discard from the cell state.

$$f_{t}=\sigma(W_{f}\cdot[h_{t-1},x_{t}]+b_{f})$$

(2) Input Gate $(i_{t},\tilde{C}_{t})$. Decides which new information to store in the cell state.

$$i_{t}=\sigma(W_{i}\cdot[h_{t-1},x_{t}]+b_{i})$$

$$\tilde{C}_{t}=\tanh(W_{C}\cdot[h_{t-1},x_{t}]+b_{C})$$

(3) Cell State Update ($C_{t}$). Updates the old cell state $C_{t-1}$ to the new state$C_{t}$.

$$C_{t}=f_{t}\odot C_{t-1}+i_{t}\odot\tilde{C}_{t}$$

(4) Output Gate $(o_{t},h_{t})$. Determines the next hidden state.

$o_{t}=\sigma(W_{o}\cdot[h_{t-1},x_{t}]+b_{o})$,

$h_{t}=o_{t}\odot\tanh(C_{t})$,

where $W$ and $b$ are weight matrices and bias vectors,$\sigma$ is the sigmoid function, and denotes element-wise multiplication.

## **Gated Recurrent Unit (GRU)**

The GRU is a simplified variant of the LSTM with fewer parameters. Its core components are the reset and update gates.

(1) Reset Gate ($r_{t}$). Determines how much of the past information to forget.

$$r_{t}=\sigma(W_{r}\cdot[h_{t-1},x_{t}]+b_{r})$$

(2) Update Gate ($z_{t}$). Controls how much information from the previous state is carried over to the current state.

$$z_{t}=\sigma(W_{z}\cdot[h_{t-1},x_{t}]+b_{z})$$

(3) Hidden State Update ($h_{t}$). The final hidden state is computed by first calculating a candidate hidden state ($\tilde{h}_{t}$) and then performing a linear interpolation between the previous state and the candidate state.

$$\tilde{h}_{t}=\tanh(W_{h}\cdot[r_{t}\odot h_{t-1},x_{t}]+b_{h})$$

$$h_{t}=(1-z_{t})\odot h_{t-1}+z_{t}\odot\tilde{h}_{t}$$

The formula symbols for the GRU are explained as follows. $t$ represents the current time step. $x_{t}$ represents the input vector at the current time step $t$. $h_{t-1}$ represents the hidden state from the previous time step $t-1$, which contains historical information. $[h_{t-1},x_{t}]$ represents the concatenation of the two vectors. $W$ represents the weight matrices for different components, which are the parameters learned by the model. $b$ represents the bias vectors for different components, which are also learned parameters. The Sigmoid activation function ($\sigma$), which outputs values between 0 and 1 and is used as a gating signal. The hyperbolic tangent activation function ($\tanh$), which outputs values between -1 and 1 and is used to generate the candidate hidden state. represents element-wise multiplication (also known as the Hadamard product).

## **Transformer**

The Transformer architecture relies solely on attention mechanisms.

(1) Scaled Dot-Product Attention. This is the core mechanism that operates on a query ($Q$), a key ($K$), and a value ($V$). The output is a weighted sum of the values, where weights are determined by the compatibility of the query and keys.

$Attention(Q,K,V)=soft\max(\frac{QK^{T}}{\sqrt{d_{k}}})V$,

where $d_{k}$ is the dimension of the key vectors, used as a scaling factor.

(2) Positional Encoding. Since the model contains no recurrence, positional encodings are added to the input embeddings to incorporate the order of the sequence. They are generated using sine and cosine functions of different frequencies:

$PE_{(pos,2i)}=\sin(pos/10000^{2i/d_{\mathrm{mod}el}})$,

$PE_{(pos,2i+1)}=\cos(pos/10000^{2i/d_{\mathrm{mod}el}})$,

where $pos$ is the position and $i$ is the dimension index.

## **Neural Ordinary Differential Equation (Neural ODE)**

The Neural ODE models the hidden state’s evolution in continuous time, making it inherently suitable for irregularly-sampled data. It assumes the derivative of the hidden state $h(t)$ can be parameterized by a neural network $f$.

(1) Initial Value Problem. The dynamics are defined by an ordinary differential equation (ODE) initial value problem:

$\frac{dh(t)}{dt}=f(h(t),t,\theta)$.

(2) Integration Solution. Given an initial state $h(t_{0})$, the state at any subsequent time $t_{1}$ is found by integrating this equation:

$h(t_{1})=h(t_{0})+\int_{t_{0}}^{t_{1}} f(h(t),t,\theta)dt$.

This integration is performed numerically using a black-box ODE solver.

## **Transformer (Pre-LayerNorm vs. Post-LayerNorm)**

Within a standard Transformer block, the relative placement of the Layer Normalization and the sub-layer (e.g., multi-head self-attention, feed-forward network) significantly impacts the model’s training dynamics and final performance. This leads to two primary architectures: Post-LayerNorm and Pre-LayerNorm.

(1) Post-LayerNorm. This is the original, standard Transformer architecture proposed by Vaswani et al. in "Attention Is All You Need". In this structure, the input signal first passes through a sub-layer, its output is then added to the original input via a residual connection, and finally, Layer Normalization is applied to the result of this sum.

$x_{out}=LayerNorm(x_{in}+Sublayer(x_{in}))$,

where $x_{in}$ is the input vector to the current Transformer block. $Sublayer(\cdot)$ represents a sub-layer operation, such as Multi-Head Attention or a Feed-Forward Network.$LayerNorm(\cdot)$ represents the Layer Normalization operation. $x_{out}$ is the final output vector of the current Transformer block.

(2) Pre-LayerNorm. This architecture places the Layer Normalization operation before the sub-layer. The input signal is first normalized, and the normalized output is then passed to the sub-layer. Finally, the sub-layer’s output is added to the original (un-normalized) input. Research has shown that this structure can effectively mitigate vanishing or exploding gradient problems, leading to more stable training, often without the need for techniques like learning rate warm-up.

$$x_{out}=x_{in}+Sublayer(LayerNorm(x_{in}))$$

In this model, all symbols have the same meaning as defined in the Post-LayerNorm section.
